# Myocardial infarction in rats was alleviated by MSCs derived from the maternal segment of the human umbilical cord

**DOI:** 10.3389/fcell.2024.1469541

**Published:** 2024-10-16

**Authors:** Shuifen Sun, Linping Wang, Qisheng Tang, Jialian Yi, Xin Yu, Yu Cao, Lihong Jiang, Jie Liu

**Affiliations:** ^1^ Regenerative Medicine Research Center, NHC Key Laboratory of Healthy Birth and Birth Defect Prevention in Western China, The First People’s Hospital of Yunnan Province, Affiliated Hospital of Kunming University of Science and Technology, Kunming, Yunnan, China; ^2^ Cell Therapy Engineering Research Center for Cardiovascular Diseases in Yunnan Province, Kunming, Yunnan, China; ^3^ Key Laboratory of Innovative Application for Traditional Chinese Medicine in Yunnan Province, Kunming, Yunnan, China; ^4^ Medicine School, Kunming University of Science and Technology, Kunming, Yunnan, China; ^5^ Department of Cardiovascular Surgery, The First People’s Hospital of Yunnan Province, Affiliated Hospital of Kunming University of Science and Technology, Kunming, Yunnan, China

**Keywords:** myocardial infarction, mesenchymal stem cells, umbilical cord, GATA4, myocd, tissue engineering

## Abstract

**Background:**

Mesenchymal stem cells (MSCs) are safe and effective in treating myocardial infarction (MI) and have broad application prospects. However, the heterogeneity of MSCs may affect their therapeutic effect on the disease. We recently found that MSCs derived from different segments of the same umbilical cord (UC) showed significant difference in the expression of genes that are related to heart development and injury repair. We therefore hypothesized that those MSCs with high expression of above genes are more effective to treat MI and tested it in this study.

**Methods:**

MSCs were isolated from 3 cm-long segments of the maternal, middle and fetal segments of the UC (maternal-MSCs, middle-MSCs and fetal-MSCs, respectively). RNA-seq was used to analyze and compare the transcriptomes. We verified the effects of MSCs on oxygen-glucose deprivation (OGD)-induced cardiomyocyte apoptosis *in vitro*. *In vivo*, a rat MI model was established by ligating the left anterior descending coronary artery, and MSCs were injected into the myocardium surrounding the MI site. The therapeutic effects of MSCs derived from different segments of the UC were evaluated by examining cardiac function, histopathology, cardiomyocyte apoptosis, and angiogenesis.

**Results:**

Compared to fetal-MSCs and middle-MSCs, maternal-MSCs exhibited significantly higher expression of genes that are associated with heart development, such as GATA-binding protein 4 (GATA4), and myocardin (MYOCD). Coculture with maternal-MSCs reduced OGD-induced cardiomyocyte apoptosis. In rats with MI, maternal-MSCs significantly restored cardiac contractile function and reduced the infarct size. Mechanistic experiments revealed that maternal-MSCs exerted cardioprotective effects by decreasing cardiomyocyte apoptosis, and promoting angiogenesis.

**Conclusion:**

Our data demonstrated that maternal segment-derived MSCs were a superior cell source for regenerative repair after MI. Segmental localization of the entire UC when isolating hUCMSCs was necessary to improve the effectiveness of clinical applications.

## Introduction

Myocardial infarction (MI) is myocardial necrosis caused by acute and persistent ischemia and hypoxia of the coronary artery and is the most common cause of death worldwide (Saparov et al., 2017; Li et al., 2022). MI leads to the death of cardiac cells and a gradual decline in heart function and eventually progresses to heart failure (Bhatt et al., 2017; Tachibana et al., 2017). While techniques such as thrombolytic therapy or coronary artery bypass surgery can restore blood flow to the heart, ischemia–reperfusion injury may cause irreversible damage (Bramucci et al., 2002; Ling et al., 2016; Guo et al., 2021). Therefore, successful treatment of MI requires rescuing dying cells, restoring blood vessels and regenerating heart tissue (Gnecchi et al., 2012; Han et al., 2015).

Stem cell-based regenerative medicine has received considerable attention for its potential in cardiac regeneration and repair. Mesenchymal stem cells (MSCs) isolated from human umbilical cord are commonly used in cardiovascular therapy due to their unique properties, including high proliferation and multilineage differentiation potential, low immunogenicity, and multiple biological functions that repair of myocardial injury after MI (Karantalis and Hare, 2015; Song et al., 2020; Zhang F. et al., 2022). Extensive preclinical studies on MSCs have demonstrated their ability to rescue dying cardiomyocytes, promote angiogenesis, reduce ventricular remodeling and infarction, protect cardiac structure, and improve cardiac function (Latifpour et al., 2011; Gnecchi et al., 2012; Zhang et al., 2013; Santos Nascimento et al., 2014; Han et al., 2015; Liu et al., 2016). However, although MSCs have shown promising therapeutic effects in preclinical animal models, the majority of registered clinical trials have not been able to achieve the desired outcomes (Levy et al., 2020; Costa et al., 2021; Zhou et al., 2021). The significant heterogeneity of MSCs has been recognized as an obstacle for clinical translation into reproducible, predictable, and standardized therapeutic approaches (Galipeau and Sensebe, 2018; Levy et al., 2020; Chen et al., 2023). Studies have shown that MSCs exhibit multifaceted heterogeneity. This heterogeneity in MSCs arises from various factors, including interindividual heterogeneity (e.g., donors, genetics, age, health status) (Hass et al., 2011), tissue-dependent heterogeneity (Du et al., 2016), different isolation methods (Juneja et al., 2016), MSC subpopulations (Zhang C. et al., 2022), and recipient cytotoxic responses (McLeod and Mauck, 2017). These differences result in a lack of consistency in MSC products, which is responsible for the poor clinical outcomes and poor reproducibility of treatments based on MSCs. Therefore, it is crucial to prioritize the standardization of MSC populations and the identification of specific MSC subpopulations to facilitate their effective translation into clinical applications (Chen et al., 2023). However, addressing the heterogeneity of MSCs and establishing a standardized approach for clinical treatment remain challenging.

The umbilical cord (UC) is a structure that connects the placenta to the developing fetus for nutrient exchange, the excretion of metabolites, and gas exchange. The UC is approximately 40–60 cm long and 1–2 cm in diameter. Previous studies demonstrated that hUCMSCs derived from maternal and fetal segments were more suitable for tissue engineering purposes in terms of cell growth, cell viability and the expression of pluripotent embryonic markers and that MSCs derived from maternal segments have greater osteogenic potential and are the preferred cell source for bone regeneration (Lim et al., 2016; Lim et al., 2018). Recently, we isolated hUCMSCs derived from the fetal, middle and maternal segments of the UC. The segment-specific cells were named fetal-MSCs, middle-MSCs and maternal-MSCs, respectively. This study examined the heterogeneous characteristics of fetal-MSCs, middle-MSCs and maternal-MSCs by examining the expression levels of genes related to cardiac development and injury repair. In addition, we further analyzed the different therapeutic effects on rats with MI.

## Materials and methods

### Isolation, culture, and characterization of hUCMSCs

Human UC samples were obtained from three healthy donors (n = 3) who delivered by cesarean section after full-term pregnancies of 38–40 weeks and provided informed consent. The use of human UC tissue was approved by the Medical Ethics Committee of the First People Hospital of Yunnan Province (KHLL2022-KY155). Three-centimeter segments from three different regions of human UCs toward the placenta (maternal segment), the center (middle segment) and toward the fetal region (fetal segment) were collected and stored in saline. MSCs isolated and cultured from 3 cm of UC tissue near the placenta are named maternal-MSCs. MSCs isolated and cultured from the central 3 cm of the entire UC length are named middle-MSCs.MSCs isolated and cultured from 3 cm of UC tissue near the fetal segment are named fetal-MSCs.Veins and arteries were removed from the UC segments, and Wharton’s jelly tissues were cut into 1–3 mm^3^ pieces and cultured as explants via the adherent method in 10-cm culture dishes containing complete medium consisting of MSC serum-free medium (MSC-T4; CSTI, Japan) and 5% UltraGRO™ (Helios, United States). After 10–15 days, the tissue explants were removed, and primary hUCMSCs (P0) were detached with CTS™ TrypLE™ Select (Gibco, United States) and cultured to passage 3 (P3). Then, hUCMSC marker identification was performed using a flow cytometer (Beckman Coulter, Chaska, MN, United States). P3 hUCMSCs were incubated with antibodies against CD44-FITC, CD90-PE, CD105-APC, CD73-APC, CD19-PerCP, CD45-FITC, CD34-FITC, CD11b-APC and HLA-DR-APC for 20 min at room temperature, and flow cytometry was performed according to the manufacturer’s instructions. All flow cytometry antibodies were obtained from BioLegend (BioLegend, United States). Finally, the adipogenic, osteogenic, and chondrogenic abilities of the hUCMSCs were assessed. P3 hUCMSCs were cultured in osteogenic, adipogenic or chondrogenic differentiation medium (Gibco, United States) according to the manufacturer’s instructions. Oil red O staining, Alizarin Red staining and Alcian Blue staining were used to evaluate adipogenesis, osteogenesis, and chondrogenesis, respectively.

### RNA-sequencing (RNA-seq)

Total RNA was extracted from UCMSCs using a TRIzol reagent kit (Invitrogen, Carlsbad, CA, United States) according to the manufacturer’s protocol. After the total RNA was extracted, mRNA was then enriched using Oligo (dT) beads. The mRNA samples were fragmented into short fragments and reverse transcribed into cDNA with random primers. Second-strand cDNA was synthesized with DNA polymerase I, RNase H, dNTPs and buffer. Then, the cDNA fragments were purified with a QiaQuick PCR extraction kit (Qiagen, Venlo, Netherlands), the ends were repaired, poly(A) was added, and Illumina sequencing adapters were ligated. The ligation products were size selected by agarose gel electrophoresis, amplified by PCR, and sequenced using an Illumina NovaSeq 6,000 by Gene Denovo Biotechnology Co. (Guangzhou, China). RNA differential expression analysis was performed with DESeq2 (4) software. Genes with a false discovery rate (FDR) less than 0.05 and an absolute fold change ≥2 were considered differentially expressed genes (DEGs). Gene Ontology (GO) biological function enrichment analysis and Kyoto Encyclopedia of Genes and Genomes (KEGG) pathway enrichment analysis were performed for all DEGs.

### Coculture of MSCs with cardiomyocytes

A transwell system (0.4-μm pore polycarbonate, Corning, United States) was used to coculture MSCs with AC16 cells. AC16 cells were purchased from the Beina Chuanglian Biotechnology Institute (Beijing, China). AC16 cells were seeded at a density of 10^5^ cells/well in 6-well plates, and MSCs were seeded at a density of 10^5^ cells/well in a transwell chamber and cultured overnight at 37°C. Then, the cells were subjected to OGD as previously described. After being replaced in glucose- and FBS-free DMEM, AC16 cells and MSCs were exposed to hypoxia (94% N_2_, 5% CO_2_, and 1% O_2_) in an anaerobic incubator (Thermo Fisher Scientific, United States) at 37°C for 72 h. Flow cytometry was used to determine the apoptotic rates of AC16 cells using an Annexin V-FITC apoptosis detection kit (Beyotime, China).

### Flow cytometric analysis of apoptosis

Flow cytometry was used to determine the apoptotic rates of AC16 cells using an Annexin V-FITC apoptosis detection kit (Beyotime, China) according to the manufacturer’s instructions. The cells were harvested using trypsin and resuspended in 195 μL of binding buffer. The cells were stained with 5 μL of Annexin V-FITC and 10 μL of PI for 15 min at 25°C. The stained cells were examined by flow cytometry (NovoCyte D2060R, Agilent Technologies, United States).

### MI model establishment and intramyocardial hUCMSCs injection

Seven-week-old male Sprague–Dawley (SD) rats were purchased from Beijing Vital River Laboratory Animal Technology Co. The rats were fed under standard animal room conditions (humidity, 55%–60%; temperature, 25°C). Food and water were freely available throughout the experiments. The animal experiments were approved by the Animal Ethical and Welfare Committee of MDKN Biotech Co., Ltd (Approval No. MDKN- 2022-068). The MI model was established by ligating the LAD as described previously. Briefly, the rats were anesthetized by isoflurane inhalation (3%–5%), endotracheal intubation was performed for mechanical ventilation, and electrocardiographic changes were recorded. A left thoracotomy was performed between the third and fourth intercostal spaces, and the LAD artery was ligated with 8–0 nylon thread. Successful ligation was verified by observing a pale left ventricular wall below the ligation site and ST-segment elevation and QRS widening via electrocardiography. The sham-operated control rats also underwent the same surgery, except that the suture placed under the left coronary artery was not tied.

Immediately after MI induction, 1 × 10^7^ hUCMSCs suspended in 50 µL of normal saline [containing 5% human serum albumin (HAS)] were injected intramyocardially at 3 different sites into the infarct border zone. Rats with MI were randomized into the following groups: 1) MI + NS group: 50 µL of normal saline (containing 5% HAS); 2) MI + fetal-MSCs group: 1 × 10^7^ hUCMSCs from the fetal segment were injected; 3) MI + middle-MSCs group: 1 × 10^7^ hUCMSCs from the central segment were injected; and 4) MI + maternal-MSCs group: 1 × 10^7^ hUCMSCs from the placenta segment were injected.

### Echocardiography

The rats were anesthetized with 3%–5% isoflurane 3 weeks after surgery. M-mode echocardiography was performed with a high-resolution Vevo 3,100 microultrasound imaging system. The left ventricular end-diastolic diameter (LVEDD) and left ventricular end-systolic diameter (LVESD) were measured on the parasternal LV long-axis view. The LVEF and LVFS were computed automatically by the echocardiography software to evaluate heart function.

### Masson trichrome staining

Masson’s trichrome staining was used to evaluate myocardial infarct size. Briefly, the myocardium was fixed in 4% paraformaldehyde (PFA, pH 7.4) and then embedded in paraffin. The heart samples were cut into three 5 μm-thick sections from the bottom to the apex of each heart. The heart sections were stained with Masson’s trichrome according to the manufacturer’s instructions. Images were scanned with a Pannoramic MIDI scanner (3D HISTECH), and the area of the infarcted zone was analyzed using ImageJ software. The infarct area was calculated as the average percentage of the fibrosis area relative to the total area.

### Immunofluorescence staining

Paraffin blocks were prepared and cut into 5 μm-thick sections. The slides were deparaffinized, dehydrated and subjected to antigen retrieval in hot citric acid buffer. The slides were permeabilized with 0.2% Triton-100 and blocked with 3% bovine serum albumin (BSA) in PBS for 1 h. The slides were incubated with CD31 primary antibodies (1:300, GB113151, Servicebio) overnight at 4°C, followed by incubation with Cy3-labeled goat anti-rabbit IgG secondary antibodies (1:300, GB21303, Servicebio) for 2 h at room temperature. Cell nuclei were stained with 4′,6-diamidino-2-phenylindole (DAPI; Servicebio) for 10 min at room temperature. Images were scanned with a Pannoramic MIDI scanner (3D HISTECH), viewed with CaseViewer software (3D HISTECH), and analyzed using ImageJ software by a double-blinded investigator. For each rat, we analyzed 6 randomly selected regions of the infarcted area of each slide at 400 × magnification.

### TUNEL staining

A Click-iT 488 TUNEL Cell Apoptosis Detection Kit (Servicebio, China) was used to examine apoptosis in myocardial sections according to the manufacturer’s protocol. Images were scanned with a Pannoramic MIDI scanner (3D HISTECH) and viewed with CaseViewer software (3D HISTECH). Six randomly selected regions in the infarction border zone of each slide were analyzed at 200 × magnification.

### Real-time quantitative PCR (qRT-PCR)

Total RNA was extracted from hUCMSCs using TRIzol reagent (Takara, Japan) according to the manufacturer’s protocols. Subsequently, the RNA was reverse transcribed to cDNA with a PrimeScript™ RT reagent kit (Takara, Japan). RT‒qPCR was performed using TB Green Premix Ex TaqTM (Takara, Japan) and a StepOnePlus real-time PCR system (Azure Biosystems). The amplification reaction conditions consisted of 95°C for 30 s, followed by 40 cycles of 95°C for 5 s and 60°C for 34 s. The sequences of the primers used were as follows: GATA4, forward: 5′-CGT​TCT​CAG​TCA​GTG​CGA​TGT​CTG-3′ and reverse: 5′-CCA​AGA​CCA​GGC​TGT​TCC​AAG​AG-3’; MYOCD, forward: 5′- CAA​CCC​TCA​CTT​TCT​GCC​CTC​ATC-3′ and reverse: 5′-GCC​ATC​GTG​TGC​TCC​TGA​GTT​C-3′; and GAPDH, forward: 5′-GCA​CCG​TCA​AGG​CTG​AGA​AC-3′, reverse: 5′- TGG​TGA​AGA​CGC​CAG​TGG​A-3′. Target gene mRNA levels were normalized to that of the reference gene GAPDH, and the relative mRNA levels were calculated using the 2^−ΔΔCT^ method.

### Western blot analysis

Protein was extracted from hUCMSCs using RIPA lysis buffer (Beyotime, China) supplemented with 1% PMSF (Biosharp, China) and quantitated using a BCA protein assay kit (TargetMol, C0001). Protein samples were separated by SDS‒PAGE and transferred to PVDF membranes (Millipore). The membranes were blocked with 5% skim milk in Tris-buffered saline with Tween 20 (TBST) at room temperature for 2 h, incubated with primary antibodies against GATA-4 (1:2000, ab256782, Abcam), MYOCD (1:2000, PA5-100775, Thermo Fisher Scientific), and GAPDH (1:10,000, AF7021, Affinity) at 4°C overnight, and subsequently incubated with the corresponding HRP-conjugated secondary antibodies (1:10,000, Affinity) for 1 h at room temperature. The bands were visualized by enhanced chemiluminescence (ECL) and quantified using ImageJ software. The housekeeping protein GAPDH was quantified for normalization.

### Statistical analysis

The data are presented as the means±SDs and were analyzed with GraphPad Prism 7 software (GraphPad Software, Inc.). Comparisons between 2 groups were performed by Student’s t-test, and multiple group comparisons were performed by one-way ANOVA. The Tukey correction was applied to account for multiple comparisons when performing *post hoc* tests. P < 0.05 was considered to indicate statistical significance.

## Results

### Maternal-MSCs expressed relatively high levels of myocardin (MYOCD), GATA-binding protein 4 (GATA4)

**FIGURE 1:**
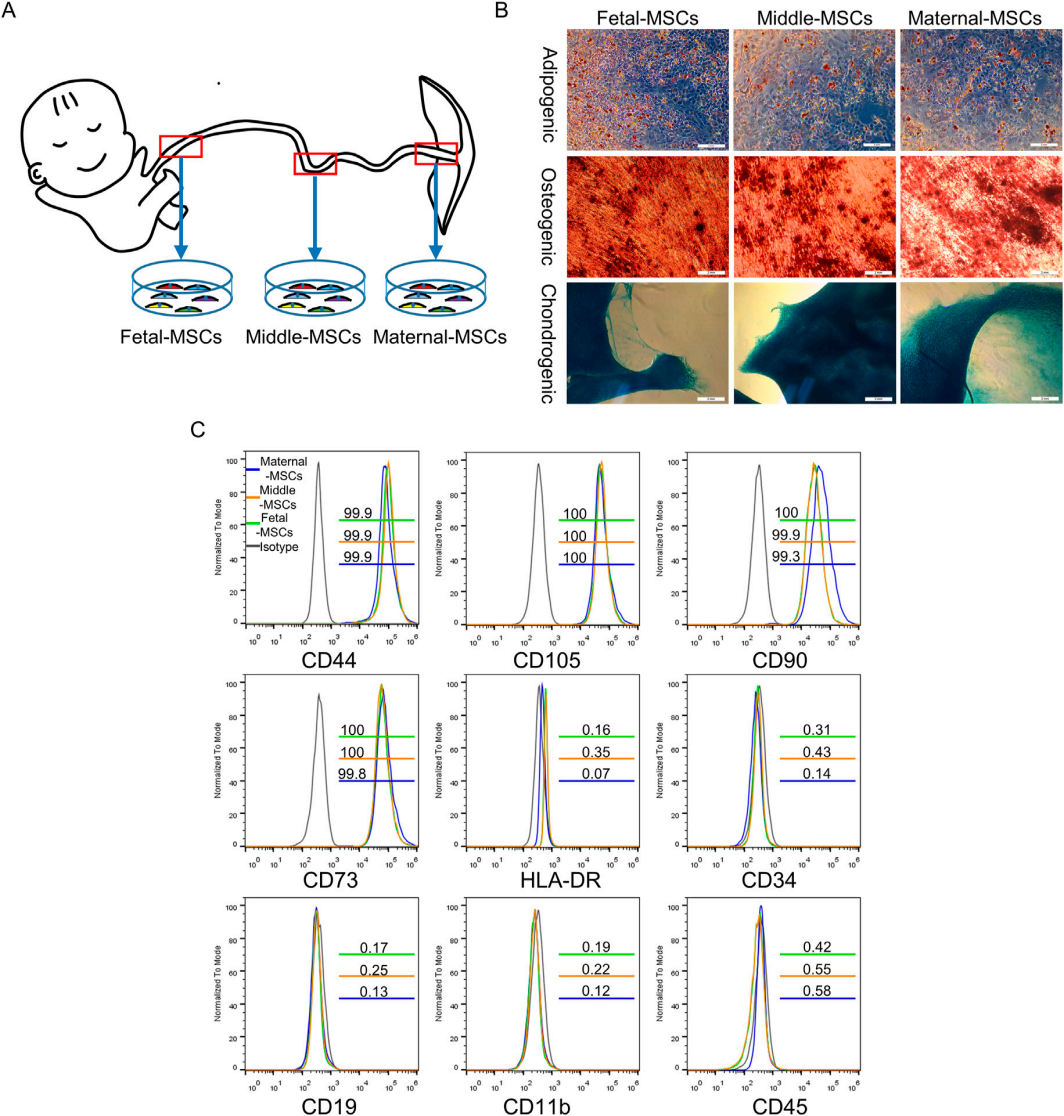
Identification of hUCMSCs. **(A)** Schematic diagram showing the isolation of MSCs derived from different segments of the human UC. **(B)** Determination of the trilineage differentiation potential of hUCMSCs. Scale bar = 2 mm. **(C)** Surface marker profiling of hUCMSCs at passage 3. The gray lines represent the isotype, the green lines represent corresponding CD marker expression in fetal-MSCs, the yellow lines represent corresponding CD marker expression in middle-MSCs, and the blue lines represent corresponding CD marker expression in maternal-MSCs.

MSCs were successfully isolated from three segments of human UCs and cultured for three passages. Flow cytometry confirmed that MSCs expressed specific mesenchymal cell markers, including CD44, CD73, CD90 and CD105 (≥95%), but lacked CD19, CD34, CD45, CD11b and HLA-DR (≤2%) (Figure 1C). Furthermore, the cells could differentiate into osteoblasts, adipocytes, and chondroblasts *in vitro* (Figure 1B). These results demonstrated that MSCs derived from different segments of the UC were successfully isolated and characterized.

**FIGURE 2:**
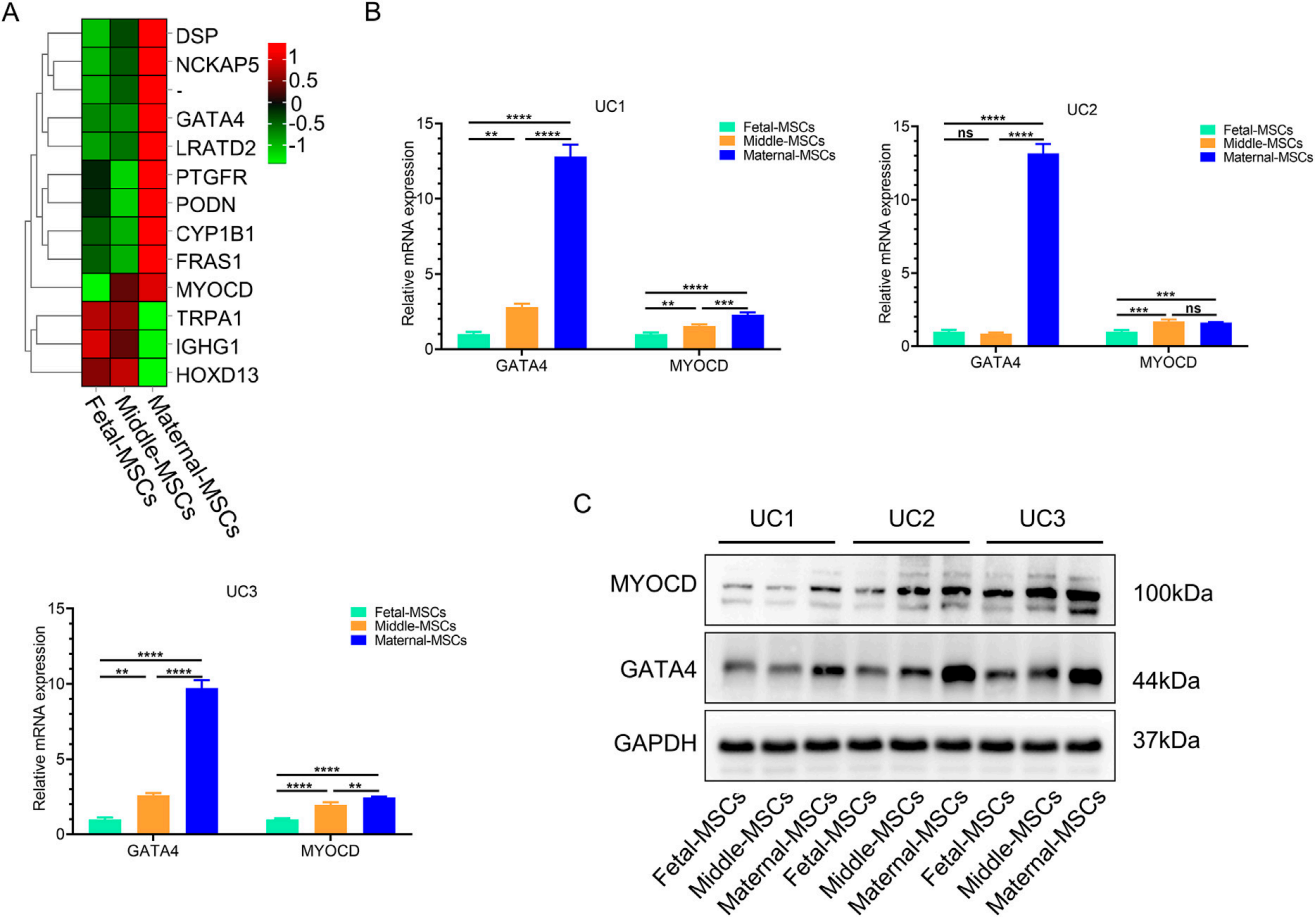
Maternal-MSCs express relatively high levels of MYOCD and GATA4. **(A)** Heatmap showing differential gene expression clustering in hUCMSCs isolated from different segments of the UC. A darker red color indicates a higher expression level, and a darker green color indicates a lower expression level. **(B)** GATA4 and MYOCD expression levels were determined by qRT‒PCR. **(C)** Representative western blot images of MYOCD and GATA4. The expression of GAPDH was used as the loading control. All the MSC experiments were performed on cells derived from three independent UCs. The data are shown as the means ± SDs. *p < 0.05, **p < 0.01, ***p < 0.001, ****p < 0.0001. GATA4, GATA-binding protein 4; MYOCD, myocardin; GAPDH, glyceraldehyde-3-phosphate dehydrogenase.

To investigate the heterogeneity of hUCMSCs isolated from different sections of the UC, we used RNA-seq technology to analyze the transcriptome and gene expression of 9 types of MSCs derived from three independent UCs. Compared to fetal-MSCs and middle-MSCs, maternal-MSCs exhibited 10 upregulated genes and 3 downregulated genes (|log2(FC)| > 1, FDR< 0.05) (Figure 2A). Maternal-MSCs highly expressed MYOCD and GATA4 (Figure 2A), which are critical transcription factors for cardiomyocyte development. These results were validated by quantitative real-time PCR analysis (Figure 2B). We further confirmed the protein expression levels of MYOCD and GATA4 by western blot analysis and showed that maternal-MSCs expressed higher protein levels of MYOCD and GATA4 than fetal-MSCs and middle-MSCs (Figure 2C). In addition, we also quantitatively analyzed the differences in GATA4 and MYOCD gene expression in hUCMSCs isolated from different segments of 7 independent UCs. The qPCR results showed that the expression levels of MYOCD and GATA4 were increased in maternal-MSCs compared to fetal-MSCs and middle-MSCs (Supplementary Figure S1). Taken together, these results demonstrated the heterogeneity of hUCMSCs isolated from different segments of the UC and the maternal-MSCs might have greater potential in cardiac protection due to higher expression of the genes associated cardiomyocte development.

### Maternal-MSCs successfully protected AC16 cells against oxygen-glucose deprivation (OGD)-induced apoptosis *in vitro*

**FIGURE 3:**
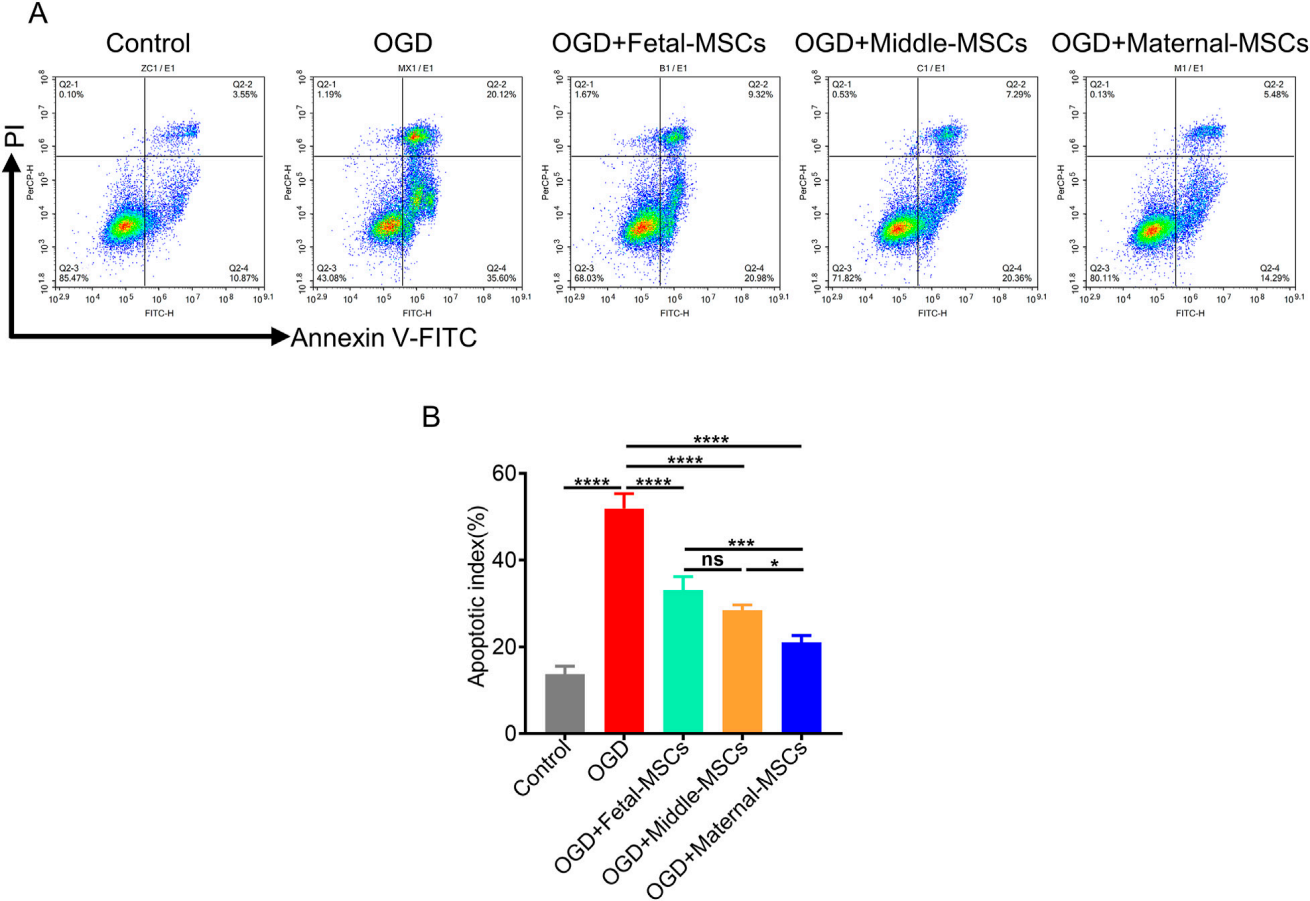
Maternal-MSCs successfully protected AC16 cells from OGD-induced apoptosis. **(A)** Representative images showing Annexin V-PI staining of AC16 cells in the five experimental groups. **(B)** Percentage of apoptotic cells in the groups (n = 3 biological replicates and n = 3 technical replicates for each biological replicate). The data are shown as the mean ± S.D. *p < 0.05, **p < 0.01, ***p < 0.001, ****p < 0.0001. OGD, oxygen-glucose deprivation.

To test the capability of MSCs derived from different segments of the UC to protect cardiomyocytes *in vitro*, AC16 cells were cocultured with fetal-MSCs, middle-MSCs and maternal-MSCs in a transwell system. Subsequently, we investigated AC16 cell apoptosis under OGD conditions. OGD resulted in significant apoptosis compared to that in the control group (Figures 3A, B). Notably, the apoptosis rate of AC16 cells was significantly lower in the maternal-MSCs group than in the fetal-MSCs and middle-MSCs groups, and there was no significant difference between the fetal-MSCs group and the middle-MSCs group (Figure 3B). The results showed that maternal-MSCs could effectively protect AC16 cells from OGD-induced cell injury *in vitro*.

### Maternal-MSCs effectively protect cardiac function in rats with MI

**FIGURE 4:**
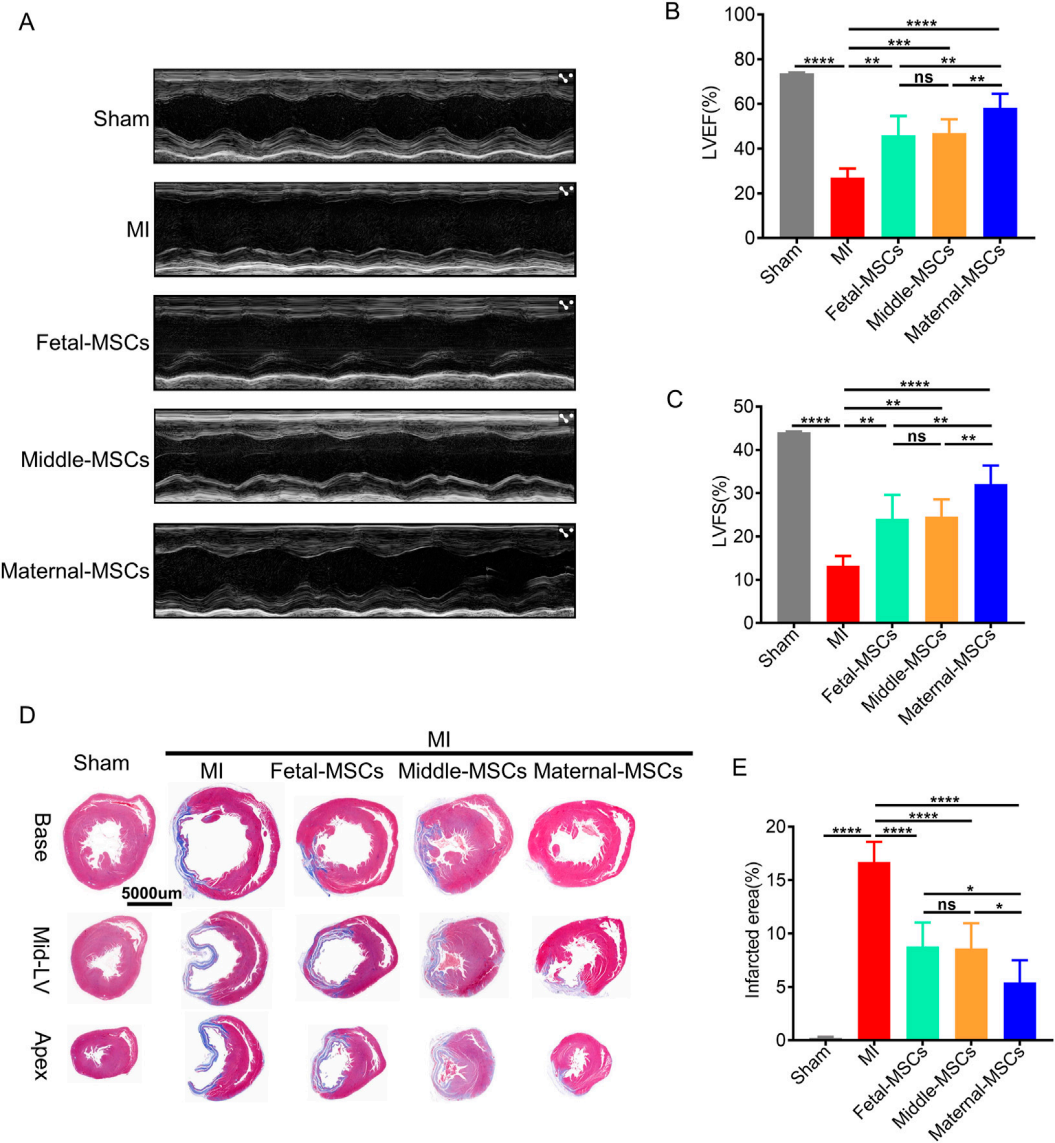
Maternal-MSCs effectively protected the cardiac function of rats with MI. **(A)** Representative echocardiographic images of rat hearts in the different groups 21 days after MI. **(B, C)** Quantitative analysis of the LVEF and LVFS 21 days after MI. **(D)** Representative Masson’s trichrome staining of the base, mid-left ventricular (mid-LV), and apical regions of an infarcted heart 21 days after MI. Scale bars: 5,000 µm. Blue indicates fibrotic tissue, and red indicates normal myocardial tissue. **(E)** Quantitative analysis of the infarct size. The data are shown as the mean ± S.D. *p < 0.05, **p < 0.01, ***p < 0.001, ****p < 0.0001. Three independent experiments were performed on hUCMSCs isolated from three different UCs,with 3 rats per group per independent experiment. MI, myocardial infarction; LVEF, left ventricular ejection fraction; LVFS, left ventricular fractional shortening.

To investigate the therapeutic effects of MSCs derived from different segments of the UC, we established an MI model in rats by permanent left anterior descending (LAD) artery ligation, after which MSCs were immediately injected intramyocardially. At 21 days after-MI, transthoracic echocardiography was performed to evaluate the *in vivo* therapeutic effects of MSCs in the rat MI model. The results showed that the left ventricular ejection fraction (LVEF) and fractional shortening (FS) of infarcted hearts were significantly lower in the MI group than in the sham group, and MSC treatment significantly rescued the MI-induced decreases in LVEF and FS (Figures 4A–C). More importantly, the maternal-MSCs group exhibited significantly higher increases in LVEF and FS than the fetal-MSCs and middle-MSCs groups (Figures 4B, C). The results indicate that maternal-MSCs exert superior protective effects on cardiac function in the rat MI model.

To determine the extent of MI-related fibrosis, Masson trichrome staining was performed 21 days after the transplantation of MSCs (Figure 4D). ImageJ analysis demonstrated that the area of fibrosis was significantly decreased in the maternal-MSCs group compared with the fetal-MSCs and middle-MSCs groups (Figure 4E). The results showed that maternal-MSCs could effectively alleviate cardiac fibrosis.

### Maternal-MSCs effectively inhibit apoptosis and promote angiogenesis in rats with MI *in vivo*

**FIGURE 5:**
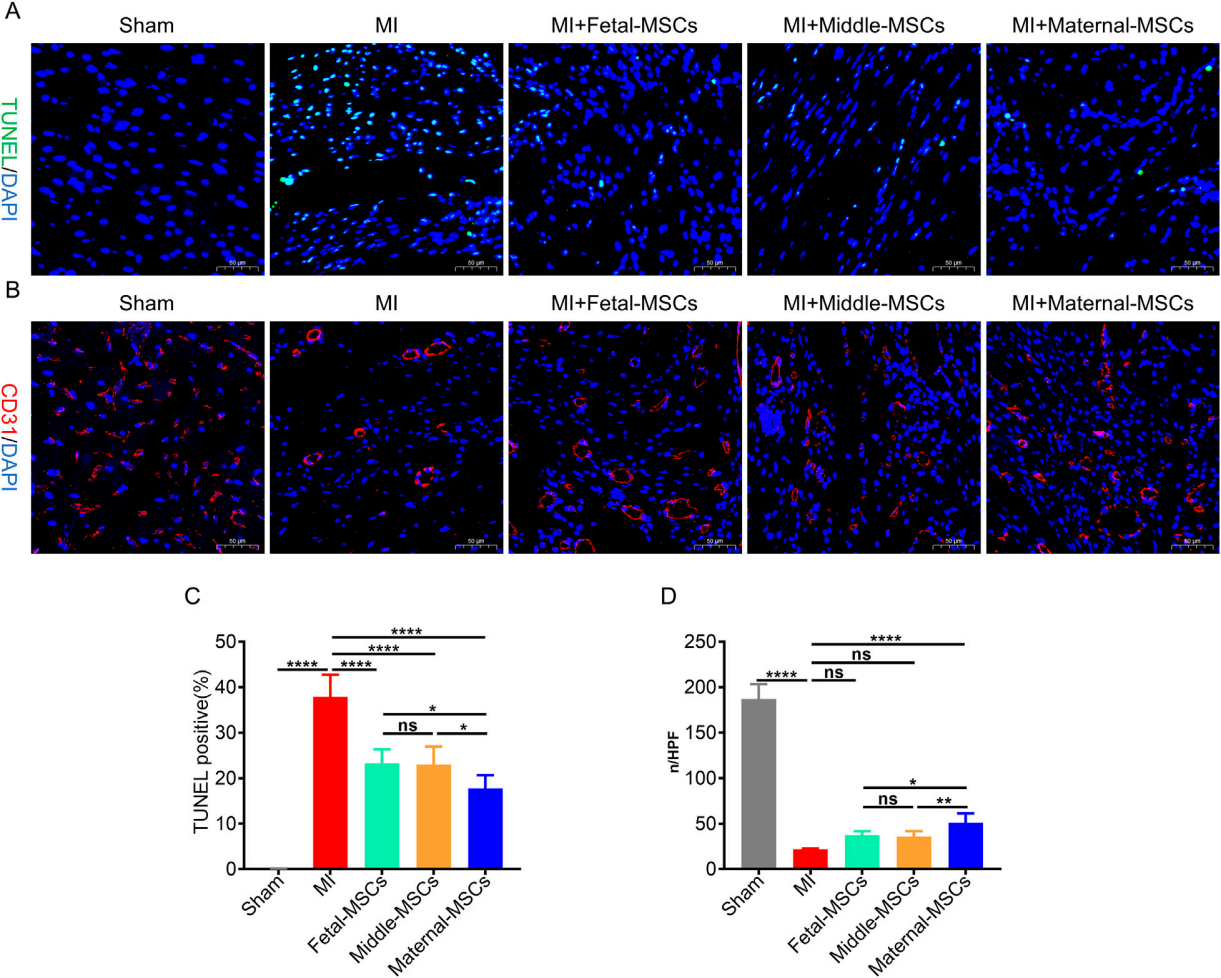
Maternal-MSCs promote angiogenesis and cardiomyocyte survival in infarcted hearts. **(A)** Representative image showing TUNEL (green) staining in the infarct border zone 21 days after MI. Nuclei are stained blue with DAPI. Scale bar = 50 μm. **(B)** Representative image showing immunofluorescent staining for CD31 (red) in the infarct and infarct border zone 21 days after MI. Nuclei are stained blue with DAPI. Scale bar = 50 μm. **(C)** Quantitative analysis of the percentages of TUNEL-positive nuclei. **(D)** Quantitative analysis of CD31-stained microvessel density. The data are shown as the mean ± S.D. *p < 0.05, **p < 0.01, ***p < 0.001, ****p < 0.0001. Three independent experiments were performed on hUCMSCs isolated from three different UCs,with 3 rats per group per independent experiment. DAPI, 4′,6-diamidino-2-phenylindole; TUNEL, TdT-mediated dUTP nick end labeling.

To evaluate the therapeutic effects of MSCs derived from different segments of the UC on angiogenesis and the inhibition of apoptosis *in vivo*, cardiomyocyte apoptosis was assessed by TUNEL staining (Figure 5A), and capillaries were stained with an antibody against CD31 and analyzed by immunofluorescence (Figure 5B). TUNEL staining revealed that the percentage of apoptotic cells in the border zone of the cardiac infarction area was significantly lower in the MSC treatment group than in the MI group (Figure 5C). Furthermore, compared with the fetal-MSCs and middle-MSCs groups, the maternal-MSCs group had significantly fewer apoptotic cells (Figure 5C). Immunofluorescence staining revealed that the capillary density in the cardiac infarction area was significantly increased in the MSC treatment groups compared with the MI group (Figure 5D). Importantly, the capillary density in the maternal-MSCs group was significantly increased compared with that in the fetal-MSCs and middle-MSCs groups (Figure 5D). These results indicated that maternal-MSCs could improve cardiac function by promoting angiogenesis and cardiomyocyte survival.

## Discussion

In the present study, we divided the UC into three anatomical segments (i.e., the maternal, middle, and fetal segments) and performed transcriptome sequencing analysis on MSCs derived from these three segments for the first time. The results showed that the cardiac transcription factors GATA4 and MYOCD were highly expressed in maternal-MSCs. We further compared the protective effects of these three MSCs on ischemic myocardial injury *in vitro* (OGD model of cultured cardimyocytes) and *in vivo* (rat model of MI) and found that MSCs derived from maternal segments showed signficantly greater protective effect in promoting the recovery of cardiac function, reducing myocardial cell apoptosis and infarct size, and increasing myocardial vessel density.

The Wharton’s jelly of the human UC has received increasing attention as a source of mesenchymal stromal cells and is now widely used in preclinical and clinical research. However, studies have shown that the cells derived from the different segments of the UC exhibit different characteristics, such as proliferative capability, viability, and osteogenic differentiation potential (Lim et al., 2016; Lim et al., 2018). To our knowledge, there has been no comparative analysis of gene expression patterns among maternal-MSCs, middle-MSCs, and fetal-MSCs isolated from different segments of the UC. In this study, we used transcriptome sequencing to determine gene expression differences among maternal-MSCs, middle-MSCs, and fetal-MSCs. The RNA-seq results showed that fetal-MSCs and middle-MSCs had similar gene expression patterns, while maternal-MSCs exhibited different gene expression pattern. What caught our attention is the expression of genes involved in cardiomyocyte development, such as GATA4, MYOCD, and DSP, was significantly increased in mat-MSCs.

GATA4 is an important transcription factor associated with heart development and heart injury repair (Rajagopal et al., 2007; Yu et al., 2016) that can regulate cell differentiation, growth and survival (Molkentin, 2000; Xu et al., 2012). Overexpression of GTAT4 not only promotes MSCs differentiation but also improves the survival of MSCs in ischemic environments, promotes angiogenesis and improves cardiac function (Li et al., 2010; Li et al., 2011). MYOCD is a muscle transcriptional cofactor with antiapoptotic and angiogenic effects (Wang et al., 2003). Downregulation of MYOCD in cardiomyocytes increases apoptosis, leading to the rapid progression of dilated cardiomyopathy and heart failure (Huang et al., 2009). Overexpression of MYOCD enhances the protective effect of MSCs against myocardial ischemia injury (Madonna et al., 2020). Since our data showed that maternal-MSCs exhibited higher expression of GATA4 and MYOCD than that of middle-MSCs and fetal-MSCs, we hypothesized that maternal-MSCs may exert superior cardioprotective effects against MI injury. Indeed, our data showed that maternal-MSCs injection improved cardiac function of the MI rats to a greater extent than the other two cells, evidenced by greater improvement in LVEF and LVFS for on echocardiography for maternal-MSCs. Moreover, left ventricular morphometric assessment by Masson trichrome staining revealed that maternal-MSCs significantly reduced the fibrotic area in the ischemic heart. These results unambiguously demonstrate that maternal-MSCs have superior protective effect against MI, however whether this effect is due to higher expression of GATA4, MYOCD in maternal-MSCs needs more experiments to verify in future studies. Although we obtained the same results from hUCMSCs isolated from UC of 7 other donors, further research is needed to determine whether maternal-MSCs from all UC overexpress GATA4, MYOCD.

MI injury leads to massive death in cardiomyocytes, therefore preventing cardiomyocyte apoptosis is an important objective of MI treatment. Previous studies have shown that MSC transplantation can effectively reduce myocardial cell apoptosis, thereby ameliorating myocardial infarction (Han et al., 2016; Liu et al., 2020). By coculturing MSCs with AC16 cells, we found that maternal-MSCs significantly reduced OGD-induced cardiomyocyte apoptosis. Our subsequent animal experiments also showed that maternal-MSCs could effectively reduce cardiomyocyte apoptosis in MI tissue. Considerable evidence suggests that MSC transplantation improves cardiac repair after MI by promoting angiogenesis (Boomsma and Geenen, 2012; Hu et al., 2016; Liu et al., 2020). We further examined the effects of MSCs derived from different segments on angiogenesis in a rat MI model. The results showed that maternal-MSCs significantly promoted angiogenesis in the rat MI model. Our study confirmed the protective effect of maternal-MSCs on cardiomyocytes in rats with MI.

## Conclusion

In summary, this study investigated the differences in gene expression among MSCs derived from three different segments of the same UC and revealed that maternal-MSCs exhibited the highest expression of genes associated with cardiomyocyte development. Furthermore, maternal-MSCs therapy significantly improved cardiac function, reduced myocardial infarct size, and exerted cardioprotective effects by inhibiting apoptosis and promoting angiogenesis in a rat model of acute MI. In conclusion, these findings emphasize the advantages of using MSCs derived from the maternal segment of the UC for repairing MI injury.

## Data Availability

The original data for this study are available from the corresponding author on reasonable request.

## Abbreviations

MSCs, Mesenchymal stem cells; UC, Umbilical cord; MI, Myocardial infarction; OGD, Oxygen-glucose deprivation; LAD, Left anterior descending; GATA4, GATA-binding protein 4; MYOCD, Myocardin; GAPDH, Glyceraldehyde-3-phosphate dehydrogenase; HAS, Human serum albumin; LVEDD, Left ventricular end-diastolic diameter; LVESD, Left ventricular end-systolic diameter; LVEF, Left ventricular ejection fraction; LVFS, Left ventricular fractional shortening; DAPI, 4′,6-diamidino-2-phenylindole; TUNEL, Terminal dexynucleotidyl transferase (TdT)-mediated dUTP nick end labeling.
