# Supplementary material for: Myocardial infarction in rats was alleviated by MSCs derived from the maternal segment of the human umbilical cord
Source: Front Cell Dev Biol. 2024 Oct 16;12:1469541. doi: 10.3389/fcell.2024.1469541 (PMC11521943; doi:10.3389/fcell.2024.1469541)
Supplement: Supplementary file 1 [file Table1.DOCX]

**Supplementary Information**


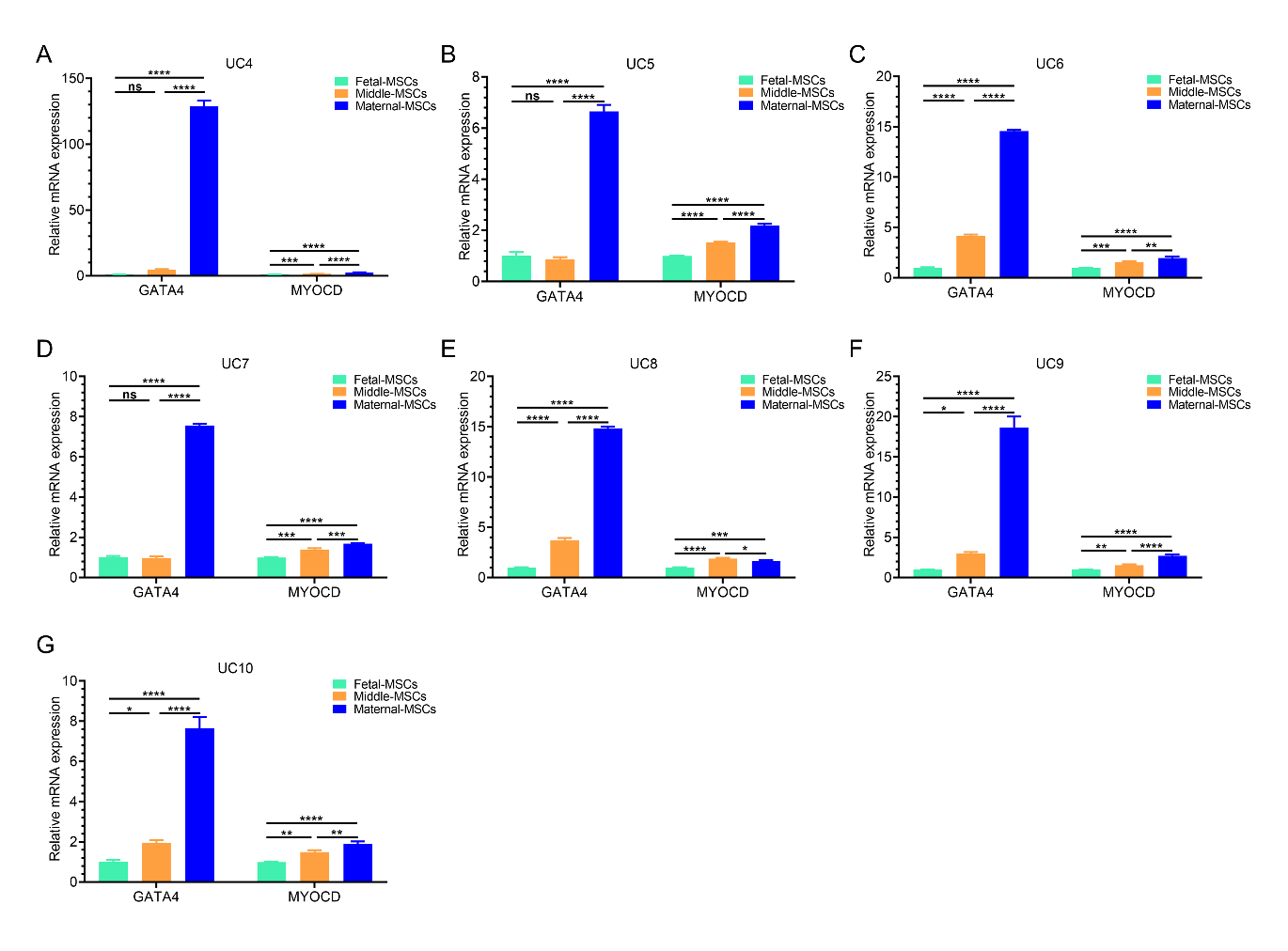
**Supplementary Figure 1.** The expression levels of the MYOCD and GATA4 genes are increased in maternal-MSCs. (A-G) The expression levels of GATA4 and MYOCD in hUCMSCs isolated from different segments of 7 independent UCs were determined by qRT‒PCR. The data are shown as the mean ± S.D. *p < 0.05, **p < 0.01, ***p < 0.001, ****p < 0.0001.
